# Current management of adults receiving oral anti-cancer medications: A scoping review protocol

**DOI:** 10.12688/hrbopenres.13208.4

**Published:** 2022-03-09

**Authors:** Janice P. Richmond, Mary Grace Kelly, Alison Johnston, Lisa Hynes, Patrick J. Murphy, Andrew W. Murphy

**Affiliations:** 1 Oncology Department, Letterkenny University Hospital, Letterkenny, Co Donegal, Ireland; 2 HRB Primary Care Clinical Trials Network Ireland, Discipline of General Practice, NUIG Galway, Galway, Ireland

**Keywords:** cancer, oral anticancer medications, care, management, assessment

## Abstract

Oncology has been undergoing a profound transition in the last ten years with the increased usage in oral anti-cancer medication. Approximately 25% of all anti-cancer medication is now designed for oral use and this is likely to increase prospectively. These treatments are convenient for patients and are often preferred by them, yet there are similar safety and toxicity concerns as there are to intravenous treatment. Oral anti-cancer medications (OAMs) have the potential to alleviate capacity issues in cancer treating units as patients receive their treatment at home, however there remains a requirement for safe and efficient assessment and care. Consequently, the management of patients on OAMs is of paramount importance. The optimum setting, whether within primary or secondary care, in addition to the appropriate health care professional to carry out patient assessment and monitoring needs to be established.

This paper presents a protocol for a scoping review which aims to systematically and comprehensively map the literature on the current management of adults receiving OAMs. The review will follow the published guidance to direct the various steps involved. The protocol will be guided by the Preferred Reporting Items for Systematic Reviews and Meta-Analyses Extension for Scoping Reviews (PRISMA-ScR) framework to ensure methodological and reporting quality. Independent full text review will be performed by two reviewers and any disagreements resolved through discussion with a third reviewer. The process will be iterative in nature.

This scoping review will provide a narrative synthesis and map the literature on the management of individuals receiving OAMs. This work is an appropriate initial stage in presenting the literature to inform the subsequent steps in a multi-phased research study which aims to establish and analyse the safety and efficacy of an integrated care model for the management of patients receiving OAM in the community by an advanced practitioner.

## Introduction

The introduction of novel oral anti-cancer medications (OAM) is a paradigm shift in cancer care and these are being approved at a record-setting pace ( Meier *et al.*, 2018) with almost 20 new approvals in oncology in the three years preceding 2019 ( U.S. Food & Drug Administration, 2018). Oral anticancer medications are a sub-set of systemic anti-cancer treatments (SACT) and have a narrow therapeutic window with a unique mechanism of action which includes pro-drugs and targeted therapies, and excludes endocrine therapy ( National Cancer Institute, 2020). The National Cancer Control Programme (NCCP) (2018) defines OAMs as all medications with direct anti-tumour activity administered by mouth (enteral route) for the treatment of cancer. They are increasingly common; a review of the NCCP website shows more than 50 different OAMs are currently in use alongside a suite of guidelines for Health Care Professionals (HCP) to direct safe and standardised patient care.

OAMs have the same benefits and risks as SACT given parenterally in terms of positive disease outcomes, treatment-related toxicities or potential for serious medication errors leading to patient harm ( NCCP, 2018). Consequently individuals receiving OAMs require frequent (at least monthly) holistic patient assessment, and serum and/or urine analysis. While these medications are convenient for individuals, it shifts the responsibility for medication management from the oncology healthcare professionals to the patient. Consequently there are concerns regarding adherence and management of toxicities or adverse effects ( Greer *et al.*, 2016; Hammond *et al.*, 2012; NCCP, 2018; Paolella *et al.*, 2018; Wood, 2012). Evidence suggests that current health care practices globally do not ensure safe administration or patient adherence ( Griffiths & Pasco, 2014; LeFebvre & Felice, 2016; Le Saux *et al.*, 2018; Redelico *et al.*, 2018; Zerillo *et al.*, 2015). A review of OAM practices in North America, Zerillo *et al.* (2015) identified unmet patient needs of 43–49% for patient education and 19–25% for adherence/toxicity monitoring. Individuals prescribed OAMs often experience a high burden of cancer related symptoms which corresponds to reduced adherence and quality of life ( Jacobs *et al.*, 2019). 

Due to the potential toxicity of these medications and associated safety challenges, the care and monitoring of patients receiving OAMs remains largely within the acute hospital setting by specialist health care teams ( Department of Health, 2017; Hammond *et al.*, 2012; Kinnaer *et al.*, 2019; NCCP, 2018). A medication safety review in Ireland noted a recurring theme of diversity and often lack of processes for the management of OAM ( Heckmann *et al.*, 2014). Furthermore, anecdotal evidence from the authors suggest that in the hospital environment individuals on OAMs receive disproportionately less input than those on parenteral treatment as the former are incorrectly perceived as being less acute and as the medication is administered at home there is less requirement to expediently progress their care at their hospital visit. To try to maximise the safety of OAMs and improve hospital efficiency, the National Cancer Strategy ( Department of Health, 2017) recommended the development of a model of care for OAM. Subsequently a guidance document for this was published by the NCCP (2018). The NCCP (2018) made 16 recommendations for the care of individuals receiving OAMs in an attempt to maximise safe practice. The focus was on safe and efficient care of individuals rather than prescriptive directions for which location that care should be delivered or by which HCP. The authors of the NCCP (2018) report recognised that there was a requirement for change within the Irish health care service to fully realise the recommendations. There is scope for these to be built upon to standardise and improve safe practice for the care of individuals receiving OAMs.

There is universal consensus in international healthcare policy documents regarding the crucial and central role which primary care should play in health care delivery. Systems with strong primary care have better health outcomes at lower costs ( House of the Oireachtas, 2017; Randall *et al.*, 2017; Starfield, 1998; World Health Organisation, 2018). Within Ireland, a shift from hospital to community-based care is being strongly promoted with the aim of delivering care closer to the person’s home within an integrated care context. This is evident in the Sláintecare health reform programme ( Government of Ireland, 2018) which endorses efforts to transform health care to maximise hospital efficiency and patient convenience. The nursing profession is recognised as critical to implementing this shift from hospital to community-based care. Specifically, nurse-led clinics for management of chronic diseases have emerged as an ideal means to achieve improved organisation in the health service ( Government of Ireland, 2018; Randall *et al.*, 2017).

Patient satisfaction levels are high with specialist or advanced practice in nursing clinics ( Liljeroos & Stromberg, 2019; Linedale *et al.*, 2020). In Ireland, Advanced Nurse Practitioners (ANP) have scope for physical examination and medicinal prescribing ( Department of Health, 2019). They have the required expertise and skills to undertake caseload management of a cohort of patients requiring advanced level decision making, such as those receiving OAMs. Currently, however advanced nursing assessment in cancer care is largely performed within a hospital context. Of particular relevance is a recent systematic review which assessed the impact of community-based nurse-led clinics on patient outcomes, and none of the studies included in the final review were oncological ( Randall *et al.*, 2017) indicating that oncology nurse-led care in the community is in its’ infancy. This review concluded that community-based nurse-led clinics have largely shown positive impact on patient outcomes, patient satisfaction and access to care despite the evidence base for such a key universal policy aim being limited. Extrapolating this to more specific care of individuals receiving OAMs, community-based care or integrated care models for this cohort of patients do not appear to be well established in national or international healthcare landscapes.

The coronavirus 2019 (COVID-19) pandemic has brought into sharp focus the requirement to reduce unnecessary hospital visits ( Cucinotta & Vanelli, 2020). This is especially pertinent to individuals receiving cancer treatment due to potential immunosuppression and concomitant risk of infection. Those patients being treated with OAM require on-going assessment and monitoring but this does not necessarily require them to repeatedly attend the hospital. Consequently, within the COVID-19 context this is an opportune time to transform the care of this cohort of patients. This would be in line with the transformative vision of a shift beyond the acute hospital, yet at the same time implement the NCCP recommendations (2018). The document by the NCCP (2018), the Sláintecare health reform programme ( Government of Ireland, 2018), the context of the COVID-19 pandemic alongside other literature promoting robust OAM patient monitoring provides the opportunity for a nurse-led integrated model of care to be developed for this patient cohort. 

**Figure 1.**
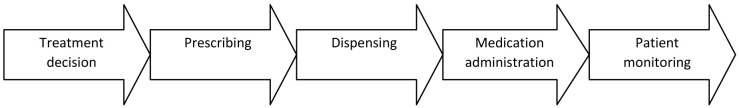
Generic systemic anti-cancer treatments (SACT) processes ( NCCP, 2018).

To consider the management of this subset of cancer patients, it is necessary to initially identify the specific aspects that constitute their assessment and care. The NCCP (2018) outlined five stages for generic SACT care which include: decision to treat, prescribing, dispensing, medication administration and patient monitoring ( Figure 1).

**Figure 2.**
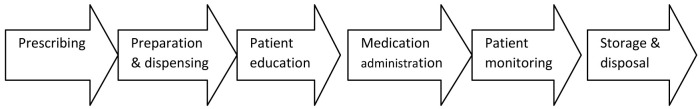
Oral anti-cancer medications (OAM) Care ( Zerillo *et al.*, 2018).

For parental SACT these stages are all performed within the hospital setting but for OAM, dispensing is performed by the community pharmacy and medication administration is within the patients’ home. Similarly Zerillo *et al.* (2018) outlines the care delivery domains for oral chemotherapy care process in their systematic review on the safety and quality of OAM. The authors do not specifically identify decision to treat as a specific stage and they include patient education occurring prior to drug administration which is a logical inclusion. Furthermore, storage and disposal are added as a final aspect of care ( Zerillo *et al.*, 2018) ( Figure 2).

For the purposes of this scoping review, the processes outlined by the NCCP (2018) and Zerillo *et al.* (2018) will be used as a framework to guide the analysis and categorisation of literature. There will be a particular emphasis on literature pertaining to prescribing, dispensing, patient education and patient monitoring as these are encompassed within the concept of patient management. Treatment decision is a baseline initial assessment based on the individuals’ suitability for treatment to commence and is not part of the daily ongoing management of care and consequently will be excluded from this review.

## Aim

A preliminary search of relevant databases, CINAHL, Medline and Web of Science was conducted, and no published systematic reviews on the overall management or continued monitoring of individuals receiving OAM were identified. Therefore, the aim of this scoping review is to identify how patients receiving OAM are currently managed from an international perspective. 

## Objectives

The specific objectives of the review are to:

- Complete a systematic search of the literature to explore the current clinical management practices for the ongoing assessment and monitoring of patients receiving OAM.
- Map existing patient management practices for those receiving OAMs with a focus on prescribing, dispensing, patient education and patient monitoring.
- From the research literature, identify any best-practice patient care recommendations that exist within current frameworks of OAM management.

This organization of information is the initial stage in a multi-phased research study which aims to establish and analyse the safety and efficacy of an integrated care model for the management of patients receiving OAM by an ANP. The scope of the review will be deliberately broad so as not to exclude the identification of best practice from any one HCP group or any country/health service. In addition, the iterative nature of this work allows for a replicable process that can be adapted to ensure it is fit for the desired endpoint. Guidance produced by Peters *et al.* (2015) will be used to direct the various stages of this scoping review. The Preferred Reporting Items for Systematic Reviews and Meta-Analyses Extension for Scoping Reviews (PRISMA-ScR) checklist will be followed throughout ( Tricco *et al.*, 2018).

### Protocol design

A search will be conducted for published and appropriate literature on the research subject. The PCC mnemonic will be used as a guide to frame the scoping review question and refers to ‘Population, Concept and Context ( Joanna Briggs Institute, 2019). In this scoping review adult cancer patients are the population, ‘oral anticancer medications’ are the concept and the context is management of care.

The three-step method for searching for studies will be applied as recommended by the Joanna Briggs Institute (2019). The databases CINAHL, Medline and Web of Science will be used for this scoping review as they collectively contain a vast range of medical, nursing and allied health literature; enabling this review to be comprehensive by capturing the majority of relevant references. The search will also include the reference lists of included papers as well as searching relevant grey literature in government reports, policy statements and conference proceedings in order to minimise the risk of publication bias. The keywords with Medical Subject Headings (MeSH) and proximity operators will be identified appropriate for each database.

An initial limited search will be conducted in these databases, followed by an analysis of text in the title and abstract, and of the index terms used to describe the article. A comprehensive search strategy will be formulated, in consultation with a university librarian, to identify relevant studies. The search strategy, including English language, identifying keywords and index terms, will be adapted for each included database as syntax of search strategies are database specific. A second search using all identified keywords and index terms will then be undertaken across all included databases. No specific search fields will be applied and all study designs will be included to keep the search broad. The third search will be of the reference list of all identified articles that are relevant to identify any potential additional further studies. Search results will be imported into a reference management software programme and duplicates removed. Any deviation from the protocol will be made clear and explained in the complete scoping review report as advocated by the Joanna Briggs Institute (2019).

### Search selection

The inclusion and exclusion criteria for this review are shown in Table 1. Only English language articles published in the years 2010–2020 will be included; justification for this being English is the vernacular of the authors and OAM are a recent development ( NCCP, 2018). The systematic review by Zerillo *et al*. (2018) of OAM safety and quality encompassed the past 20 years but all included articles were published after 2007 with 75% (n = 12) in the last 3 years. The authors note that the types of OAM medications have become more diverse over time and most of the earlier publications focused on capecitabine in patients with breast or colorectal cancers. Therefore, as this scoping review aims to consider contemporary management of care for this patient population who are receiving all OAMs it is sufficient to focus on studies published from 2010–2020 inclusive.

**Table 1.** Inclusion and exclusion criteria.

Inclusion criteria
English language articles
Articles published 2010–2020
Adults: individuals of 18 years old or more
• Allow inclusion of relevant studies with mean age over 18 years of age
Studies pertaining to the ongoing management of adults receiving oral anti-cancer medication.
Exclusion criteria
Non-human studies

The search terms that are to be used are outlined in Table 2 using the PCC. Three reviewers (JR, MGK, AJ) will independently review the retrieved articles for inclusion based on title and abstract. The articles selected at this stage will then undergo a further independent full text review by the reviewers (JR, MGK) to determine relevance. Any disagreement will be resolved through discussion with a third reviewer (AJ). As quality assessment does not form part of a scoping review our study will not include assessment of methodological quality of the included papers ( Arksey & O'Malley, 2005). Throughout the scoping review however, the authors will review and report the salient aspects of the studies reviewed. This will include the quantity of the different types of articles reviewed and will allow descriptive expansion on the items listed in the data extraction table below ( Table 3), in particular the methodologies, study populations, sample size, HCP involved and models of care utilised as this has direct relevance to the subject under review.

**Table 2.** Search terms used for scoping review of the current Management of Cancer Care for Patients Receiving OAMs.

Population:	Concept:	Context:
(Cancer) patients	Oral anticancer medicine	Cancer Care management
Cancer	Oral anticancer*	Care
Oncolog*	(Oral anticancer drug*)	Management
Neoplasm*	(Oral anticancer agent*)	Model*
Patient*	(Oral anticancer medic*)	Clinic*
	(Oral anticancer therap*)	Unit*
(NOT child/paediatric/adolescent)	(Oral anticancer treatment*)	(Health) Service*
	Oral antineoplastic*	“Delivery of health care”
	(Oral antineoplastic drug*)	
	(Oral antineoplastic agent*)	
	(Oral antineoplastic medic*)	
	(Oral antineoplastic therap*)	
	(Oral antineoplastic treatment*)	
	Oral chemotherap*	
	(Oral chemotherapeutic drug*)	
	(Oral chemotherapeutic agent*)	
	(Oral chemotherapeutic medic*)	
	Medication therapy management	

### Data extraction

Data extraction will be performed by two reviewers independently. Microsoft Excel 2010 will be used for the management of the screening, duplicate removal and data extraction stages of the scoping review. Endnote will be used for reference management. A data extraction table ( Table 3) will be used to capture the characteristics relating to the aims and objectives of the scoping review. Pre-testing with a pilot of up to five studies will be performed by three reviewers (JR, MGK, AJ) to evaluate the appropriateness and suitability of the headings used in the data extraction tool. The data extraction table will structure the review of each study and enable the authors to standardise the reporting of work reviewed. Furthermore, it will focus the authors to search for and report on the relevant aspects of each study to ensure detailed and accurate review. This is especially pertinent when more than one individual is performing the review and facilitates the sharing of findings from the scoping review between the research team members.

**Table 3.** Definitions for data extraction.

Descriptive term	Definition for the purpose of the scoping review
Author(s)	Name of individual (s) writing the paper
Year	Year paper was published
Country/countries	Country/countries in which the paper was published
Publication type	List whether it is paper, conference presentation, website or other
Aims	List the aims of the paper
Type of study/methodology	List the method used to gather the data-e.g. Randomised controlled trial, ethnographic, interventional, descriptive etc.
Study population	Type of malignant pathology that the paper primarily focuses on for the study population • Select if the cancer(s) studied is haematological (non-solid) or oncology (solid) or both. • For solid malignancies select the best option to describe the primary cancer as breast/colorectal/prostate/gastric/variety of cancers or unspecified
Sample size	Number of participants in the study
HCP primarily involved	The HCP primarily involved in the study (e.g. nurse/medical/pharmacist/variety)
Model of care identified	Is a defined or established framework for delivery care identified?
Patient monitoring	Is on-going scheduled monitoring/assessment of patients on OAMs described or outlined?
Recommendation(s)	Identify any recommendation(s) for improvement of patient care on OAMs

### Data analysis

Descriptive numerical summaries and narrative synthesis, that aligns with the scoping review aim and objectives, will be presented in appropriate formats e.g. maps, tables. Narrative synthesis refers to an approach to the systematic review and synthesis of findings from multiple studies that relies primarily on the use of words and text to summarise and explain the findings and utilises a textual approach to ‘tell the story’ of the findings from included studies ( Popay *et al.*, 2006). Search results, selection process results, additions from reference searching, etc. and the final number of included sources will be presented in a PRISMA-ScR flow chart.

### Dissemination of information

The result of the scoping review is critical as a means to gather the relevant evidence base and inform further aspects of the study. As the research team plan to produce a nurse-led community model for care of individuals on OAMs, the scoping review will provide an overview of the current management of this cohort of patients and identify essential aspects of care required for safe practice. Furthermore, any recommendations for practice identified by existing literature will be included to enable the model of care developed by this work to conform to best practice standards. Once the scoping review has been completed, the findings will be disseminated in two ways. Firstly the results will be presented to a study advisory panel, consisting of the collaborators and invited national experts to discuss models of potential processes and outcomes, and produce a proposal for an ANP integrated oncology care model in the community. Furthermore, the results will be disseminated by publication in a peer-reviewed journal.

### Study status

At the time of publication of this protocol, informal preliminary searches of the literature have been commenced primarily to help to identity key search terms.

## Discussion

The scoping review protocol is an essential component in the process of performing a scoping review of a chosen topic ( Moloney *et al.*, 2020) and provides an outlined method for exploring and mapping relevant literature ( Lafferty *et al.*, 2019). Individuals being treated with OAM require on-going assessment and monitoring ( NCCP, 2018) yet with increasing numbers of patients on OAMs there is a requirement to improve hospital efficiency and reduce overcrowding in cancer units yet provide safe, appropriate and timely patient care. The knowledge obtained in this scoping review will be presented to an advisory panel and will help inform the subsequent steps in a multi-phased research study which aims to establish and analyse the safety and efficacy of an integrated care model for the management of patients receiving OAM in the community by an ANP.

## Data availability

### Underlying data

No data are associated with this article.
