# Supplementary material for: Current management of adults receiving oral anti-cancer medications: A scoping review protocol
Source: HRB Open Res. 2022 Mar 9;4:7. Originally published 2021 Jan 28. [Version 4] doi: 10.12688/hrbopenres.13208.4 (PMC8802151; doi:10.12688/hrbopenres.13208.4)
Supplement: Peer review history

# Peer review: Reviewer response for version 4

**DOI:** 10.21956/hrbopenres.14757.r31626

**Published:** 2022-03-09

**Authors:** Melanie Dalby

**Affiliations:** 1 Barts Health NHS Trust, London, UK

I have no further comments to make.

Is the study design appropriate for the research question?

Yes

Is the rationale for, and objectives of, the study clearly described?

Yes

Are sufficient details of the methods provided to allow replication by others?

Partly

Are the datasets clearly presented in a useable and accessible format?

Not applicable

Reviewer Expertise:

Cancer pharmacy, oral anti-cancer medications, patient experience, cancer in the community, oncology, haematology.

I confirm that I have read this submission and believe that I have an appropriate level of expertise to confirm that it is of an acceptable scientific standard.

# Peer review: Reviewer response for version 3

**DOI:** 10.21956/hrbopenres.14733.r31491

**Published:** 2022-02-24

**Authors:** Melanie Dalby

**Affiliations:** 1 Barts Health NHS Trust, London, UK

I am happy to amend the status to Approved. I have just one further comment, please remove the word ‘between’ in **bold** below:

*‘This is especially pertinent when more than one individual is performing the review and facilitates the sharing of findings **between** from the scoping review between the research team members’*

Is the study design appropriate for the research question?

Yes

Is the rationale for, and objectives of, the study clearly described?

Yes

Are sufficient details of the methods provided to allow replication by others?

Partly

Are the datasets clearly presented in a useable and accessible format?

Not applicable

Reviewer Expertise:

Cancer pharmacy, oral anti-cancer medications, patient experience, cancer in the community, oncology, haematology.

I confirm that I have read this submission and believe that I have an appropriate level of expertise to confirm that it is of an acceptable scientific standard.

# Peer review: Reviewer response for version 2

**DOI:** 10.21956/hrbopenres.14485.r31164

**Published:** 2022-01-28

**Authors:** Melanie Dalby

**Affiliations:** 1 Barts Health NHS Trust, London, UK

This study protocol has been well prepared and written. I just have a few comments for you to consider.

1. It would be useful if you could include the search terms you are planning to use.
2. Sentence starting: ‘Furthermore, anecdotal evidence from the authors … less input that those…’ please change that to than.
3. I understand that a quality assessment of each paper will not be completed but will you report on the quantity of different article types i.e. number of randomised controlled trials, observational studies, etc.?
4. For the aim can you add whether this is locally, nationally, or internationally?
5. Please can you explain how the data extraction table will be used in a bit more detail?
6. Sentence: ‘…indicating that oncology nurse-led care in the community is in its’ infancy.’ Can you remove the apostrophe?
7. Sentence starting: ‘The search strategy, including English language, identified keywords …’ I think it would read better if you said ‘identifying’ rather than ‘identified’, please have a look.

Is the study design appropriate for the research question?

Yes

Is the rationale for, and objectives of, the study clearly described?

Yes

Are sufficient details of the methods provided to allow replication by others?

Partly

Are the datasets clearly presented in a useable and accessible format?

Not applicable

Reviewer Expertise:

Cancer pharmacy, oral anti-cancer medications, patient experience, cancer in the community, oncology, haematology.

I confirm that I have read this submission and believe that I have an appropriate level of expertise to confirm that it is of an acceptable scientific standard, however I have significant reservations, as outlined above.

# Peer review: Reviewer response for version 2

**DOI:** 10.21956/hrbopenres.14485.r29740

**Published:** 2021-07-14

**Authors:** Eileen Furlong, Andrew Darley

**Affiliations:** 1 School of Medicine, University College Dublin, Dublin, Ireland; 2 School of Nursing, Midwifery and Health Systems, University College Dublin, Dublin, Ireland

Thank you for considering and including our suggestions and comments.  We have no further comments to make on this revised article.

Is the study design appropriate for the research question?

Yes

Is the rationale for, and objectives of, the study clearly described?

Yes

Are sufficient details of the methods provided to allow replication by others?

Partly

Are the datasets clearly presented in a useable and accessible format?

Not applicable

Reviewer Expertise:

Cancer care; patient outcomes; integrated care; scoping reviews

We confirm that we have read this submission and believe that we have an appropriate level of expertise to confirm that it is of an acceptable scientific standard.

# Peer review: Reviewer response for version 1

**DOI:** 10.21956/hrbopenres.14360.r29033

**Published:** 2021-03-30

**Authors:** Paolo Tralongo

**Affiliations:** 1 Medical Oncology Unit, Ospedale Umberto I, RAO, Siracusa, Italy

The paper on the protocol proposed belongs to a fascinating and important issue in the oncology field and daily debated.

1. The rationale and objectives of the study are clearly described. However, the aim of making any recommendations for the clinicians should undergo to a valid and shared by scientific community method, to assess and grading quality of evidence and strength of any potential recommendation.
2. The study design appears appropriate for the research question. However I would modify the exclusion criteria, they should not be only the opposite of inclusion criteria.
3. I would suggest to gather and summarize the findings of the studies included, categorizing them by the quality and strength of the evidence, through the type of studies (i.e. randomized controlled trial, observational studies, etc..).
4. The manuscript is extremely concise and it does not provide all details of the proposed research.

Is the study design appropriate for the research question?

Partly

Is the rationale for, and objectives of, the study clearly described?

Partly

Are sufficient details of the methods provided to allow replication by others?

Yes

Are the datasets clearly presented in a useable and accessible format?

Yes

Reviewer Expertise:

Medica oncologist

I confirm that I have read this submission and believe that I have an appropriate level of expertise to confirm that it is of an acceptable scientific standard, however I have significant reservations, as outlined above.

# Peer review: Reviewer response for version 1

**DOI:** 10.21956/hrbopenres.14360.r28770

**Published:** 2021-02-17

**Authors:** Eileen Furlong, Andrew Darley

**Affiliations:** 1 School of Medicine, University College Dublin, Dublin, Ireland; 2 School of Nursing, Midwifery and Health Systems, University College Dublin, Dublin, Ireland

Thank you for the opportunity to review your submission. Oral anti-cancer medication is a very pertinent healthcare issue for advanced nurse practitioners and this scoping review is very timely in light of the initiative towards enhancing integrated care. The protocol is well-written and the reviewers have some comments for consideration.

Abstract

- In the first sentence specify “increase" regarding oral anti-cancer medications, i.e., *availability or usage.*
- Consider rewording and clarifying the points in the last sentence in the first paragraph of the Abstract i.e., “patients on oral anti-cancer”, “as to the location of such patient assessment”.
- Use “protocol” rather than report.
- Last sentence: suggest using “advanced nurse practitioner” for consistency. 

Introduction

- Please specify the concept in this sentence “The nursing profession is recognised as critical to implementing this.”

Aim/Objectives

- Clearly state the research question of the scoping review.
- Consider changing “This approach” to “The knowledge obtained in this review could…”
- Please specify the patient management practices as mentioned in the Objectives. 

Methodology

- Are you using a screening tool e.g., Covidence?
- How will study selection be recorded? Is this being done through Excel or are the authors using a reference manager?
- Will duplicates be removed manually by the author team?
- In the abstract it states that the review will be performed by two reviewers and checked by a third reviewer, however on P8 that 'any disagreement will be resolved through discussion with a third reviewer'. Please confirm the process.
- Provide more specific detail on “relevant grey literature” and justify why it is being included.  It would be helpful if the authors provided the search strategy for the grey literature and how it will be limited. e.g. search terms, sources. 
- Consider using initials of the author team when referring to the reviewers.
- Statement regarding “desired endpoint” – consider using “replicable” process rather than repetitive.
- Consider removing “crucially” in Protocol Design.
- Consider changing “natural language” to “English”.
- Reword sentence beginning with “To keep the search broad..."
- Did the authors consider using SCOPUS in their search?
- Will the authors conduct a quality appraisal on the identified studies? If not, provide justification.
- Consider including Limitations of the studies reported in Table 2, which will help analysis.
- Please use “A nurse-led…” not “an”.

Conclusion

- Consider reframing the final two sentences to address the value of this scoping review in light of the aims and objectives.

Is the study design appropriate for the research question?

Yes

Is the rationale for, and objectives of, the study clearly described?

Yes

Are sufficient details of the methods provided to allow replication by others?

Partly

Are the datasets clearly presented in a useable and accessible format?

Not applicable

Reviewer Expertise:

Cancer care; patient outcomes; integrated care; scoping reviews

We confirm that we have read this submission and believe that we have an appropriate level of expertise to confirm that it is of an acceptable scientific standard, however we have significant reservations, as outlined above.
